# Metagenomic Analysis of a Tropical Composting Operation at the São Paulo Zoo Park Reveals Diversity of Biomass Degradation Functions and Organisms

**DOI:** 10.1371/journal.pone.0061928

**Published:** 2013-04-24

**Authors:** Layla Farage Martins, Luciana Principal Antunes, Renata C. Pascon, Julio Cezar Franco de Oliveira, Luciano A. Digiampietri, Deibs Barbosa, Bruno Malveira Peixoto, Marcelo A. Vallim, Cristina Viana-Niero, Eric H. Ostroski, Guilherme P. Telles, Zanoni Dias, João Batista da Cruz, Luiz Juliano, Sergio Verjovski-Almeida, Aline Maria da Silva, João Carlos Setubal

**Affiliations:** 1 Departamento de Bioquímica, Instituto de Química, Universidade de São Paulo, São Paulo, Brazil; 2 Departamento de Ciências Biológicas, Universidade Federal de São Paulo, São Paulo, Brazil; 3 Escola de Artes, Ciências e Humanidades, Universidade de São Paulo, São Paulo, Brazil; 4 Instituto de Computação, Universidade Estadual Campinas, Campinas, Brazil; 5 Fundação Parque Zoológico de São Paulo, São Paulo, Brazil; 6 Departamento de Biofísica, Escola Paulista de Medicina, Universidade Federal de São Paulo, São Paulo, Brazil; 7 Virginia Bioinformatics Institute, Virginia Tech, Blacksburg, Virginia, United States of America; AC Camargo Cancer Hospital, Brazil

## Abstract

Composting operations are a rich source for prospection of biomass degradation enzymes. We have analyzed the microbiomes of two composting samples collected in a facility inside the São Paulo Zoo Park, in Brazil. All organic waste produced in the park is processed in this facility, at a rate of four tons/day. Total DNA was extracted and sequenced with Roche/454 technology, generating about 3 million reads per sample. To our knowledge this work is the first report of a composting whole-microbial community using high-throughput sequencing and analysis. The phylogenetic profiles of the two microbiomes analyzed are quite different, with a clear dominance of members of the *Lactobacillus* genus in one of them. We found a general agreement of the distribution of functional categories in the Zoo compost metagenomes compared with seven selected public metagenomes of biomass deconstruction environments, indicating the potential for different bacterial communities to provide alternative mechanisms for the same functional purposes. Our results indicate that biomass degradation in this composting process, including deconstruction of recalcitrant lignocellulose, is fully performed by bacterial enzymes, most likely by members of the Clostridiales and Actinomycetales orders.

## Introduction

Decomposition of organic matter in a typical composting process is carried out by a complex microbial community whose structure changes depending on temperature, pH, aeration, water content, and type and amount of organic solids [1]–[6]. The aerobic microbial metabolism drives pH changes and rapid temperature increase above 50°C, followed by sustained high temperatures between 60–80°C and then gradual cooling of the composting mass [7].

Analyses of different composting environments by cultivation-dependent or community fingerprinting by amplified rDNA restriction analysis, denaturing gradient gel electrophoresis (DGGE), DNA hybridization techniques and phospholipid fatty acid determination have shown that Actinomycetales, Bacillales, Clostridiales and Lactobacillales are among major bacterial orders identified in composting processes [6], [8]–[12]. For instance Lactobacillales have been associated with the initial mesophilic stage in the composting of organic household waste, which often has a low initial pH [2], [6], [9]. On the other hand, Bacillales, Clostridiales and Actinomycetales have been shown to constitute a substantial part of the community in the thermophilic stages of composting of organic household waste [6], [10] or a mixture of livestock manure and shredded plant waste [8], [11]. In addition a few fungal species have been also identified among compost microbial communities during its thermophilic stage as well as upon cooling [1], [13], [14].

The above mentioned composting studies were focused on the detection of abundant microbial groups and limited by biases imposed by rRNA gene-cloning or probing approaches [15]–[18]. These limitations could potentially be overcome by advances in DNA extraction protocols [19] and sequencing technologies [20]–[22] as well as by computational methods for whole-community sequence data analysis [22]–[24], which together allow a comprehensive overview of the phylogenetic composition and diversity of genes in complex microbial communities. For instance, metagenomic approaches are guiding discovery of enzymes and organisms for biomass deconstruction using samples from complex environments such as cow and yak rumen [25]–[27] and switchgrass-adapted compost [20], [28], [29].

Here we present analyses of a large data set (1.6 Gbp) generated by direct pyrosequencing of metagenomic DNA from composting samples, with the goal of investigating their microbial community composition and to prospect for genes and functions related to biomass degradation. Samples were collected at a composting facility inside the São Paulo Zoo Park, which is located within the urban area of the São Paulo megalopolis (Brazil), and includes a significant remnant patch of Atlantic rain forest. The composting facility is designed to compost four tons/day of all organic waste produced in the park. Dropped tree leaves, plant debris and grass clippings collected from the Atlantic rain forest fragment and gardens located inside the park, water recycling slurry from its artificial lake, waste water treatment sludge, bedding materials and animal feed wastes, plus animal excrements from about 400 species are blended and composted by a standardized management procedure in several 8 m^3^ open concrete chambers, followed by stabilization in windrows (unpublished procedure). The end compost humus-rich material obtained after 80–100 days is used as fertilizer and soil amendment in the São Paulo Zoo Farm, thus completing the full cycle of recycling. About 600 tons of compost end product is generated per year. The hypothesis that guided our study was that given its peculiar composition, the São Paulo Zoo Park compost process would host a large microbial diversity, combining the phylogenetic richness of soil and forest microbial communities [30]–[32] with that of the microbiota associated with zoo animals [33]–[35]. To our knowledge this work is the first report of high-throughput sequencing and analysis of a composting whole-DNA microbial community.

## Results and Discussion

### Shotgun Pyrosequencing of Compost Microbiomes

To assess the microbial diversity and the metabolic potential for biomass degradation in the composting process from the São Paulo Zoo we applied a sequence-based metagenomic approach. Samples were collected during the composting operation, one from a chamber 8 days after the beginning of composting process (Zoo Compost 1, ZC1) and another from a chamber 60 days after the beginning of composting process (Zoo Compost 2, ZC2); the latter had been thoroughly mixed and aerated eight days before sampling. In both operations the total composting time was about 90 days. High molecular weight DNA extracted from samples ZC1 and ZC2 was submitted to shotgun sequencing using the Roche 454 GS FLX Titanium technology. Four sequencing runs yielded over 2,900,000 reads per sample with 276 and 299 nt mean length, totaling 836 Mbp and 842 Mbp, for ZC1 and ZC2, respectively (Table 1). Assembly of these two metagenomic sequence datasets yielded 52,953 contigs for ZC1 and 52,182 contigs for ZC2, each one using, respectively, 37.2% and 48.8% of the total reads. N50 contig length of 1,734 bp and 1,516 bp was obtained for ZC1 and ZC2, respectively.

**Table 1:** 454 GS FLX Titanium pyrosequencing and Newbler assembly metrics of two metagenomic DNA samples from São Paulo Zoo composting.

Parameter	Zoo Compost 1	Zoo Compost 2
Total number of reads	3,167,044	2,966,244
Mean read length	276 nt	299 nt
Metagenome size (unassembled reads)	836 Mbp	842 Mbp
Metagenome size(assembled reads)	506.0 Mbp	433.7 Mbp
Number of reads in contigs	1,178,578 (37.2%)	1,448,502 (48.8%)
Number of contigs	52,953	52,182
Reads/contig	22.26	27.76
Largest contig (bp)	39,861	65,988
Mean contig length (bp)	1,384	1,332
N50 contig length (bp)	1,734	1,516
Number of singletons	1,842,944	1,404,679

**Figure 1:**
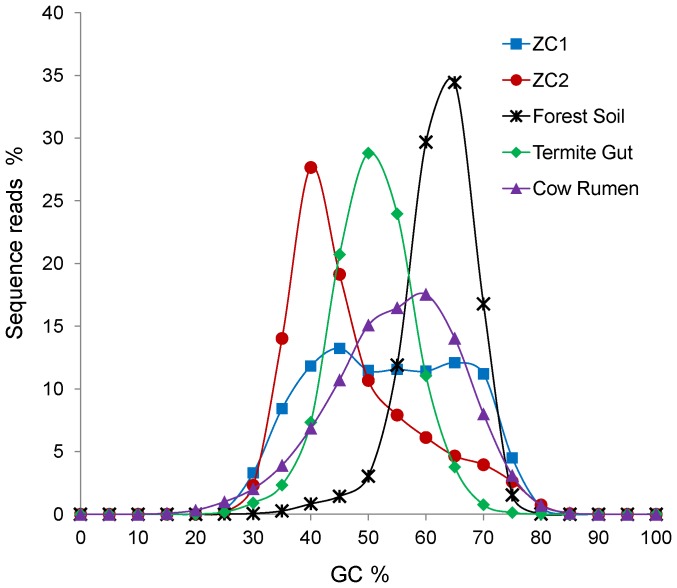
Distribution of the GC content percentage for ZC1 and ZC2 compared with selected metagenomes. Each position represents the percentage of sequences reads within a GC percentage range. Sources: ZC1 and ZC2 (this work); Luquillo Experimental Forest Soil at Puerto Rico [36]; termite gut [37] and cow rumen pooled planktonic [25] metagenomes were retrieved from MG-RAST.

The ZC1 metagenome exhibits average GC content higher than ZC2 (Table 2) and its sequence reads also present a very distinct GC content profile when compared with ZC2 (Fig. 1). Besides differing between themselves in GC content, both ZC1 and ZC2 are also markedly different in GC content from three publicly available high-throughput sequencing datasets related to biomass degradation (soil from a Puerto Rico rain forest, termite gut and cow rumen planktonic microbiomes [25], [36], [37]) (Fig. 1), suggesting differences in their respective microbial composition [38], [39], which is supported by results shown below.

**Table 2:** Features of the composting metagenomes based on MG-RAST^a^ and IMG/M^b^ annotations.

Annotation Platform	MG-RAST		IMG/M	
Metagenome/Features	ZC1	ZC2	ZC1	ZC2
Total number of reads post MG-RAST quality control	2,200,727	2,019,033	–	–
Total DNA scaffolds post IMG/M data processing	–	–	1,720,157	1,373,328
Average GC content	51±12%	45±11%	–	–
Protein coding sequences	2,512,832	2,366,522	1,512,472	1,257,499
Protein coding sequences with function prediction	1,373,548 (54.7%)	1,438,584 (60.8%)	857,144 (56.2%)	732,661 (57.8%)
Protein coding sequences with enzyme classification(EC) prediction	**ND**	**ND**	359,301 (23.6%)	317,233 (25.0%)
rRNA genes	13,352	15,832	4,131	3,569

a Features from unassembled reads that passed MG-RAST quality control.

b Features from Newbler assembled reads post IMG/M data processing.

**ND, not determined.**

### Compost Microbial Community Composition

Overall community structure analyses performed with M5RNA (Non-redundant multisource ribosomal RNA annotation) and M5NR (M5 non-redundant protein) databases available within MG-RAST [40] showed that ZC1 and ZC2 are dominated by species in the Bacteria domain (84–89% and 93–96%, respectively), regardless of the database used (Table S1). The remaining sequences match Archaea (<1%), Virus (<0.25%) and Eukaryota (<3%) sequences, or were unassigned. The few Eukaryota rRNA sequences found in both samples are mostly related to Streptophyta, Nematoda, and Arthropoda phyla and possibly correspond to residual DNA from the compost start substrate. We observed that the fraction of ZC1 and ZC2 protein-coding sequences related to fungi was negligible (less than 0.02% of all reads in either sample).

**Figure 2:**
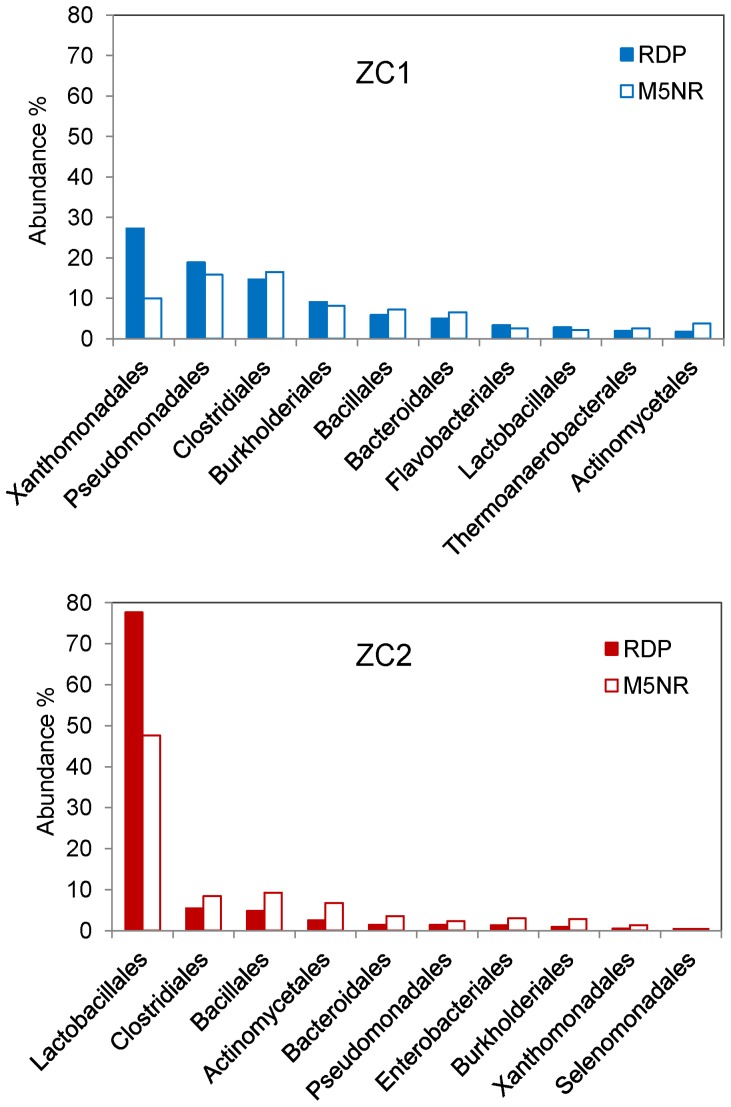
Microbial Community Composition of ZC1 and ZC2 metagenomes. Unassembled reads annotated on MG-RAST were analyzed using the classification tool based on RDP (98% identity; e-value cutoff of 10^−30^) and M5NR (60% identity; e-value cutoff of 10^−5^) with minimum alignment length of 50 bp. The figure displays the taxonomic distribution for the 10 most abundant orders.

The Bacteria domain composition of ZC1 and ZC2 metagenomes was further investigated using the RDP [41] and M5NR databases available within MG-RAST [40]. Despite the striking differences in abundance, most bacterial orders found in both samples (Table S2) are among major bacterial classes previously identified in composting processes [6], [8], [9], [11], [12], [42]–[44]. (The baseline for all fractions reported henceforth refer to all reads assigned to the Bacteria domain.) Proteobacteria is by far the most abundant phylum in ZC1 (58% and 48% according to RDP and M5NR, respectively), while Firmicutes dominates the ZC2 bacterial community (88% and 67% according to RDP and M5NR, respectively). The ten most abundant orders in ZC1 and ZC2 bacterial communities are shown in Figure 2. The observed difference in abundance is significant (*p*<0.01) as determined by the RDP library compare tool using the Naive Bayesian classifier [45]. While in ZC1 75% of the total bacterial orders are represented by Xanthomonadales, Pseudomonadales, Clostridiales, Burkholderiales and Bacillales, in ZC2 ∼75% are solely represented by Lactobacillales. This high abundance of Lactobacillales might reflect the more advanced stage of the compost process of the ZC2 sample relative to ZC1 or unknown characteristics of the ZC2 initial composting substrate. In contrast, an early work by rRNA cloning and sequencing has shown that members from the lactic acid bacteria were present during the initial stages of composting in a model bench-scale reactor system, and their presence correlates with low pH in the feeding and mesophilic composting conditions [46]. In our case, the observed differences could not be correlated with pH or temperature, since at the moment of sampling temperatures were 66°C and 67°C for ZC1 and ZC2, respectively, and pH was 7.0 for both samples.

Despite the fact that the composting process such as the one we prospected here is an aerobic process, we found a noteworthy abundance of Clostridiales (∼15% in ZC1; ∼6% in ZC2), which is a bacterial order known to include anaerobic or micro-aerophilic species. This probably reflects the semi-static conditions of the compost we sampled, which favors the formation of anaerobic micro-environments, and also the high metabolic activity of the bacterial community [1], [6], [7], [47]. Anaerobic microorganisms have been proposed to play an important role in biomass degradation [47], [48] and, indeed, *Clostridium* appears to be responsible for cellulose degradation in composting [1], [11], [49], [50]. Therefore, the appearance of Clostridiales since the initial stages of composting seems important for degradation of complex biopolymers such as hemicellulose and cellulose.

Degradation of complex polymers in compost appears to be performed also by Actinomycetales, Bacillales and fungi, whose presence has been associated with age and temperature of composting [1], [6]. In these studies Actinomycetales have been shown to be abundant in thermophilic stages, while fungi appear towards the end of the composting process, in the cooling and maturation phase. Even though fungi are well-known agents of lignocellulose degradation, cumulative evidence suggests that members from Actinomycetales and Bacillales among other bacterial orders possess the ability to degrade cellulose and solubilize lignin [48], [51]. Moreover, they tolerate higher temperatures and higher pH than fungi, and usually colonize the substrate once the less complex carbon sources have been exhausted [1], [6], [52]–[55]. Our results show that, despite their relatively low sequence abundance, Actinomycetales and Bacillales (respectively, 1.8% and 5.9% in ZC1; 2.5% and 4.9% in ZC2) are among the 10 top bacterial orders in our compost samples, which were both collected at thermophilic stages. These results are in line with cultivation-dependent observations showing *Bacillus* among the dominant bacterial *taxa* recovered from compost during the thermophilic phase [1].

**Figure 3:**
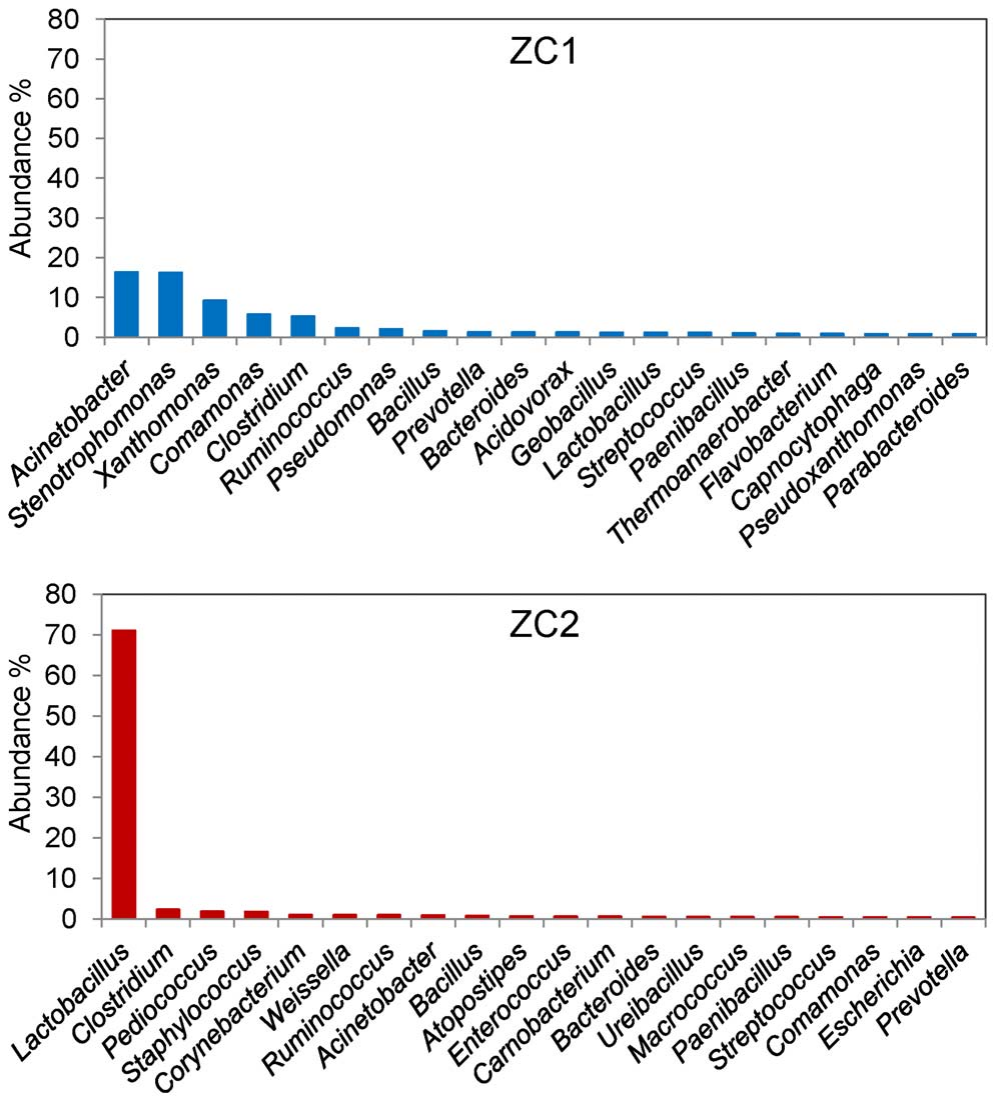
Most abundant bacterial genera in ZC1 and ZC2 compost samples. Unassembled reads annotated on MG-RAST were analyzed using the classification tool based on RDP (98% identity; e-value cutoff of 10^−30^; minimum alignment length of 50 bp). The figure displays the taxonomic distribution for the 20 most abundant bacterial genera.

ZC1 and ZC2 16S-rRNA reads were further taxonomically classified at the level of genus by means of the RDP Naive Bayesian Classifier [45] (Fig. 3). In ZC1 the five most abundant genera are *Acinetobacter*, *Stenotrophomonas*, *Xanthomonas*, *Comamonas* and *Clostridium*, which account for more than half (∼52%) of all identified genera, while in ZC2 about 70% of the 16S-rRNA sequences were assigned to genus *Lactobacillus*. An analysis performed with the M5NR database also shows similar results (data not shown). The remaining bacterial community in both samples appears to be distributed in more than two hundred different genera (Table S3).

**Figure 4:**
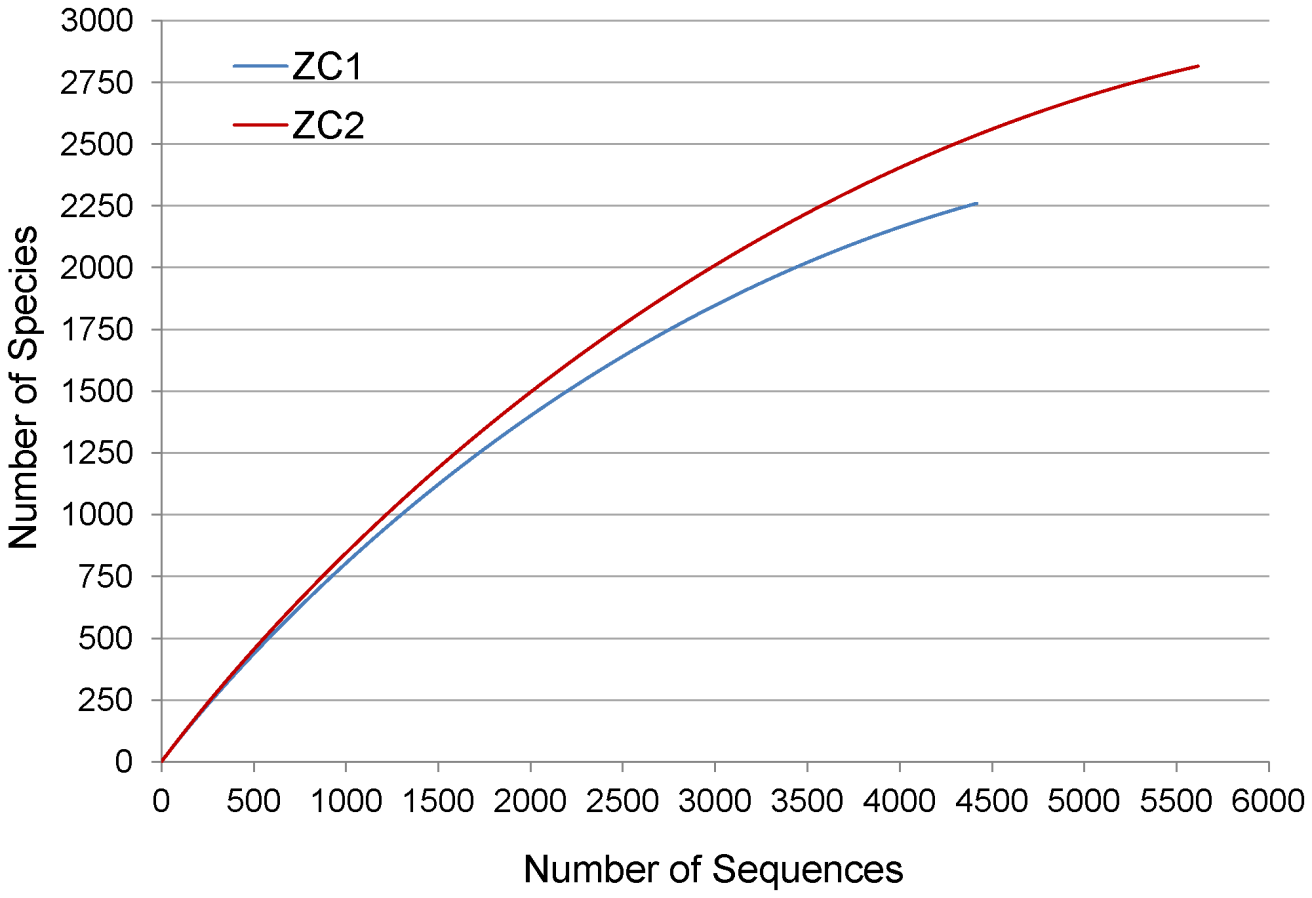
Rarefaction curves for ZC1 and ZC2 metagenomes. rRNA-related sequences were retrieved from the whole metagenomic data set and classified on RDP to obtain rarefaction curves at genetic distance of 3%.

Rarefaction curves from the samples were determined at genetic distance of 3% by using rRNA-related sequences retrieved from the whole metagenomic sequences dataset (4,420 sequence reads for ZC1 and 5,616 sequence reads for ZC2). The rarefaction curves (Fig. 4) did not reach saturation, with the number of species sampled being 2,260 and 2,816 for ZC1 and ZC2, respectively. These numbers are lower bounds on the species richness of the two samples and they support our initial hypothesis that the Zoo composting process would host a large microbial diversity. We do not report diversity estimators as given by indexes such as Chao1or Shannon because such estimators are strongly biased by sample sizes and do not seem to yield reliable results [56].

### Species Diversity of Lactobacilli in ZC2

As discussed above the genus *Lactobacillus* predominates in the ZC2 metagenome (Fig. 3). This result is consistent with previously reported results from a recent study of the microbial diversity of a composting process in pilot and full-scale operations performed in drum units fed with organic municipal waste [6]. There are other studies reporting presence of Lactobacilli in composting [8], [57]–[59]. In the Partanen et al. study [6], based on analyses of 1,560 reads generated from 16S rRNA gene libraries from 18 samples, *Lactobacillus* was found to be highly abundant at the start of the process (reaching more than 90% in one of the samples, 4 days into the composting process [6]). The presence of *Lactobacillus* in these samples correlated with low pH (4.7–5.9) and mesophilic temperatures, except for one sample where pH was 7.8 [6]. This contrasts to some extent with the ZC2 sample conditions, which had thermophilic temperatures and pH 7.0. Presence of *Lactobacillus* under thermophilic conditions is consistent with previous reports [60], [61].

The genus *Lactobacillus* encompasses over 140 species with a high degree of genetic diversity [62], [63]. The diversity of *Lactobacillus* in ZC2 was additionally explored by comparing its unassembled reads to 16S rRNA, nucleotide, and protein sequence databases. These analyses predicted the presence of at least 45 *Lactobacillus* species (Table S4), which is indicative of the remarkable diversity of this genus in ZC2. The most abundant *Lactobacillus* species in ZC2 were *L. brevis* (26.5%), *L. plantarum* (3.4%), *L. oris* (3.4%), *L. johnsonii* (3.3%), *L. amylovorus* (3.2%), and *L. fermentum* (2.8%).

Lactobacilli are almost ubiquitous and found in environments where carbohydrates are available such as dairy products, fermented fish and sourdoughs [64]–[66]. As members of the lactic acid bacteria (LAB) group, a number of *Lactobacillus* species are recognized as safe bacteria and are used as probiotics and/or starter cultures in food and feed fermentation [62], [67]. Due to their competitiveness and adaptation to the environmental conditions, certain LAB species dominate specific fermentation processes, and it is believed that production of bacteriocins plays an important role in this competitive advantage [61], which might justify the dominance of *Lactobacillus* in the ZC2 metagenome. Moreover, the ZC2 sample was collected after 60 days of composting, when most of the hemicelluloses and cellulose have been converted to less complex carbohydrates, allowing colonization by thermophilic Lactobacilli. A recent study [68] identified *Lactobacillus* species in the feces of 16 animals classified as carnivores, omnivores and herbivores. *L. johnsonii* and *L. reuteri* were among the most abundant species isolated from carnivores (though also present in omnivore and herbivore feces), and *L. plantarum*, *L. brevis* and *L. casei* were isolated from omnivores. Such results are consistent with our observations of *Lactobacillus* diversity in ZC2 and the use of diverse animal fecal material in the ZC2 composting substrate.

### Functional Profiling of Compost Metagenomes

**Figure 5:**
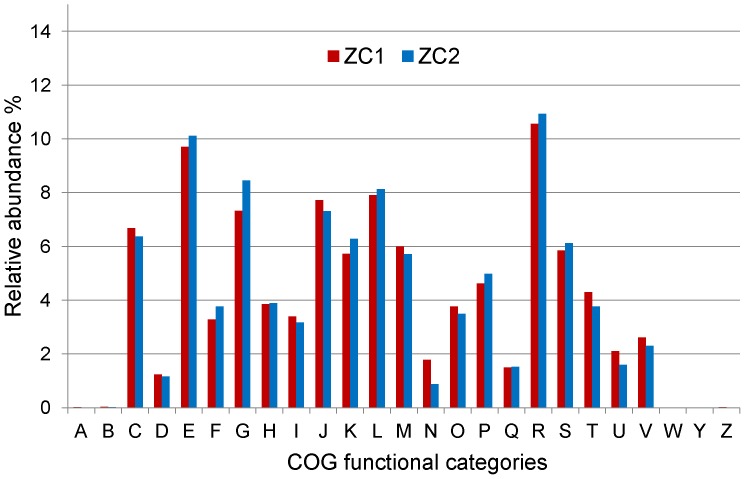
Relative abundance of COG functional categories for ZC1 and ZC2 metagenomes. Assembled sequence reads were classified into the 25 COG functional categories, and their relative abundances for ZC1 and ZC2 metagenomes were estimated considering the total number of protein coding sequences with function prediction. Designations of functional categories: A: RNA processing and modification, B: Chromatin structure and dynamics, C: Energy production and conversion, D: Cell cycle control, cell division, chromosome partitioning, E: Amino acid transport and metabolism, F: Nucleotide transport and metabolism, G: Carbohydrate transport and metabolism, H: Coenzyme transport and metabolism, I: Lipid transport and metabolism, J: Translation, ribosomal structure and biogenesis, K: Transcription, L: Replication, recombination and repair, M: Cell wall/membrane/envelope biogenesis, N: Cell motility, O: Posttranslational modification, protein turnover, chaperones, P: Inorganic ion transport and metabolism, Q: Secondary metabolites biosynthesis, transport and catabolism, R: General function prediction only, S: Function unknown, T: Signal transduction mechanisms, U: Intracellular trafficking, secretion, and vesicular transport, V: Defense mechanisms, W: Extracellular structures, Y: Nuclear structure, Z: Cytoskeleton.

The functional profiles of the ZC1 and ZC2 metagenomes were determined by classification of predicted genes based on Clusters of Orthologous Groups (COG/KOG) [69] assignments. At the highest level of the COG category system, ZC1 and ZC2 exhibit a similar profile (Fig. 5). Moreover, ZC1 and ZC2 exhibit approximately the same COG functional categories distribution seen in general for prokaryotes [69], reflecting the dominance of the Bacteria domain in these microbiomes. As expected, typical eukaryotic KOG functional categories (RNA processing and modification, Chromatin structure and dynamics, Extracellular structures, Cytoskeleton and Nuclear structure) are not represented in our sequence data set.

Functional specificities of ZC1 and ZC2 are revealed using deeper levels of the COG hierarchy. Among assigned COG functions we observed many that are relevant to the expected characteristics of a complex microbial community engaged in biodegradation. For instance, some of the abundant COG functions in ZC1 and/or ZC2 (Table 3), such as hydrolases and dehydrogenases (COG1012, COG1960, COG1028, COG0673 and COG0561) and proteins involved with carbohydrate transport and metabolism (COG0395, COG1175, COG1129, COG1109, COG2814 and COG2723), can be related directly to the dynamics and recycling power in the microbial community structure in a biomass degrading environment. In addition, among the most abundant functions present in ZC1 and/or ZC2 metagenomes (Table 3), we found several COGs associated with bacterial efflux pumps (COG1132, COG0841, COG0534, COG1131 and COG1136), which are known to export substances such as antibiotics and toxic molecules [70]. We hypothesize that ZC1 and ZC2 proteins with these functions may play a role in bacterial defense against toxic metabolites such as antibiotic compounds and anti-microbial peptides, produced by many bacteria (e.g. acid lactic bacteria, *Staphylococcus* and *Bacillus)* during the composting process [57]. The 30 most abundant COG functions (Table 3) also include functions related to regulation in response to environmental stimuli such as histidine kinases and response regulators (COG0642 and COG0745) and transcriptional regulators (COG1609 and COG0583). The high proportion of these COGs could be indicative of the need to respond to the constant changes in the composting environment and to the interactions required by its microbial community.

**Table 3:** Top 30 (by sequence count) COG functions represented among ZC1 and ZC2 metagenomic assembled sequences.

			ZC1			ZC2	
COG category	COG ID	COG Name	sequence count	ranking	sequence count		ranking
V	COG1132	ABC-type multidrug transport system, ATPase and permease components	3846	1	3471		1
TK	COG0745	Response regulators consisting of a CheY-like receiver domain and awinged-helix DNA-binding domain	2935	2	2706		2
V	COG0841	Cation/multidrug efflux pump	2877	3	1892		16
V	COG0534	Na+-driven multidrug efflux pump	2578	4	1533		27
P	COG2217	Cation transport ATPase	2526	5	2608		3
C	COG1012	NAD-dependent aldehyde dehydrogenases	2442	6	2421		6
G	COG0395	ABC-type sugar transport system, permease component	2425	7	1054		64
T	COG0642	Signal transduction histidine kinase	2410	8	2037		11
V	COG1131	ABC-type multidrug transport system, ATPase component	2369	9	1995		12
O	COG0542	ATPases with chaperone activity, ATP-binding subunit	2359	10	2395		8
I	COG1960	Acyl-CoA dehydrogenases	2517	11	2259		31
K	COG1609	Transcriptional regulators	2259	12	2517		5
J	COG0480	Translation elongation factors (GTPases)	2236	13	1572		25
IQ	COG0318	Acyl-CoA synthetases (AMP-forming)/AMP-acid ligases II	2212	14	1861		17
L	COG0178	Excinuclease ATPase subunit	2164	15	2162		10
IQR	COG1028	Dehydrogenases with different specificities (related to short-chain alcohol dehydrogenases)	2169	16	2146		9
L	COG0188	Type IIA topoisomerase (DNA gyrase/topo II, topoisomerase IV), A subunit	2131	17	1925		14
L	COG0587	DNA polymerase III, alpha subunit	2040	18	1749		19
R	COG0488	ATPase components of ABC transporters with duplicated ATPase domains	2005	19	2599		4
M	COG0768	Cell division protein FtsI/penicillin-binding protein 2	1996	20	1459		30
G	COG1175	ABC-type sugar transport systems, permease components	1993	21	1035		69
L	COG0187	Type IIA topoisomerase (DNA gyrase/topo II, topoisomerase IV), B subunit	1990	22	1671		22
K	COG0583	Transcriptional regulator	1960	23	2403		7
L	COG4974	Site-specific recombinase XerD	1883	24	1812		18
L	COG0210	Superfamily I DNA and RNA helicases	1815	25	1426		32
J	COG0621	2-methylthioadenine synthetase	1750	26	906		93
M	COG0438	Glycosyltransferase	1732	27	1509		28
G	COG1129	ABC-type sugar transport system, ATPase component	1729	28	889		95
R	COG0673	Predicted dehydrogenases and related proteins	1686	29	1226		44
G	COG1109	Phosphomannomutase	1683	30	1315		35
V	COG1136	ABC-type antimicrobial peptide transport system, ATPase component	1673	33	1569		26
E	COG0436	Aspartate/tyrosine/aromatic aminotransferase	1617	36	1625		24
P	COG0474	Cation transport ATPase	1380	44	1894		15
C	COG1249	Pyruvate/2-oxoglutarate dehydrogenase complex, dihydrolipoamide dehydrogenase (E3) component, and related enzymes	1334	50	1681		21
EH	COG0028	Thiamine pyrophosphate-requiring enzymes [acetolactate synthase, pyruvate dehydrogenase (cytochrome), glyoxylate carboligase, phosphonopyruvate decarboxylase]	985	96	1470		29
G	COG2814	Arabinose efflux permease	694	262	1940		13
R	COG0561	Predicted hydrolases of the HAD superfamily	561	369	1726		20
G	COG2723	Beta-glucosidase/6-phospho-beta-glucosidase/beta-galactosidase	367	665	1642		23

793758 and 680461 annotated assembled sequences of ZC1 and ZC2 metagenomes were respectively classified in 4529 and 4872 COGs and ranked according to their abundance. Sequence count and ranking for COGs outside the top 30 list are indicated in numbers with smaller font.

The ZC1 set includes a group of predicted genes annotated as coding for cellulase M and related proteins (COG1363 and EC 3.2.1.4). An alignment of two of these ZC1 predicted protein sequences (349 and 350 aa) with *Clostridium thermocellum* cellulase M results in 50% identity (Figure S1). Despite the difficulty in distinguishing CelM from the M42 family of peptidases based on sequence similarity [71], *C. thermocellum* CelM shows endoglucanase activity and appears to be noncellulosomal [72]. The ZC1 metagenome includes other predicted genes related to cellulose degradation activities in higher abundance when compared with the ZC2 metagenome. For instance, while the ZC1 metagenome has 112 predicted protein sequences annotated as cellulase (glycosyl hydrolase family 5) and 32 predicted protein sequences annotated as proteins with cellulose binding domain, ZC2 has only 19 and two sequences, respectively, with the same annotation. In addition, we were able to identify 65 predicted protein sequences containing the dockerin domain (pfam00404) and 36 predicted protein sequences with the cohesin domain (pfam00963) in the ZC1 metagenome. Although in much lower abundance, the ZC2 metagenome also contains predicted genes annotated with these functions, six and eight sequences with the dockerin and cohesin domains, respectively. These enzymes and protein modules are known components of the cellulosome, a multienzyme complex that mediates the deconstruction of hemicellulosic substrates by anaerobic bacteria [73]. Accordingly, 867 predicted genes annotated with COG3459 (cellobiose phosphorylase EC:2.4.1.20), for an enzyme family that is key for microbial cellulose utilization [48], are found in the ZC1 metagenome, while ZC2 contains 267 such sequences.

**Figure 6:**
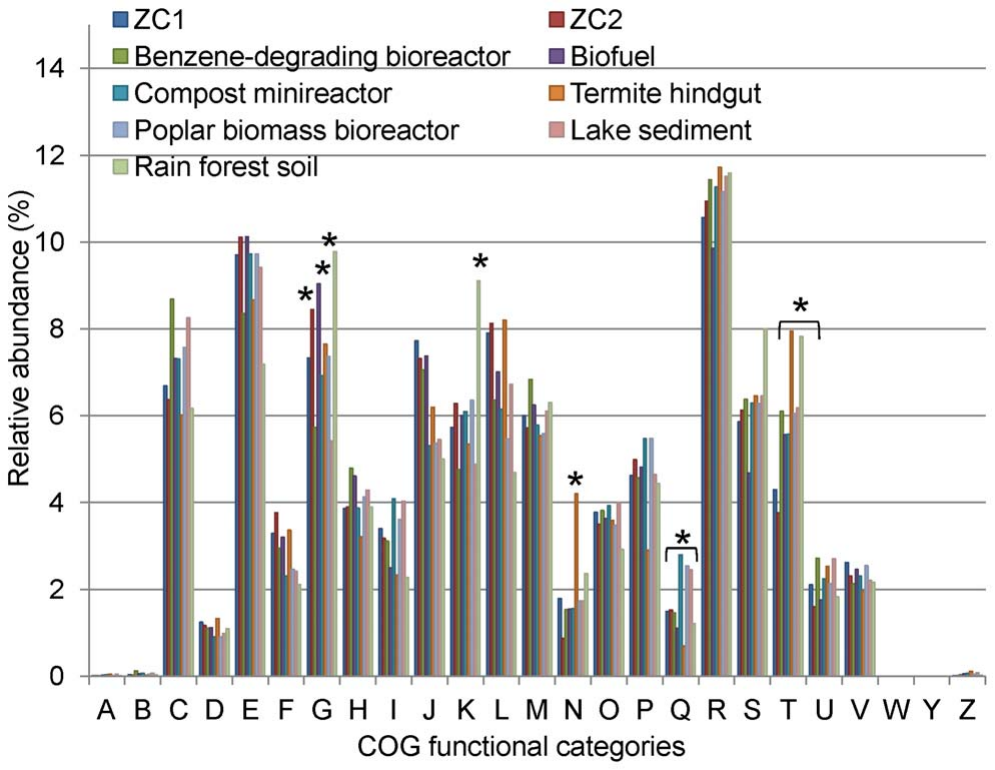
ZC1 large contig encoding pectin degradation enzymes. ZC1 sequences assembled into a 27,919 bp contig encoding the following proteins: 1. Beta-xylosidase (376 aa, COG3507); 2. Dehydrogenases (280 aa, COG1028); 3. hypothetical protein (379 aa); 4. hypothetical protein (283 aa); 5. 5-keto 4-deoxyuronate isomerase (280 aa, COG3717); 6. Dehydrogenases (267 aa, COG1028);7. hypothetical protein (1799 aa); 8. SusD family protein (606 aa, pfam07980); 9. TonB-linked outer membrane protein (1068 aa, COG4771); 10. Pectate lyase (518 aa, COG3866); 11. Predicted unsaturated glucuronyl hydrolase (398 aa, COG4225); 12. Pectin methylesterase (568 aa, COG4677); 13. Endopolygalacturonase (523 aa, COG5434); 14. Nucleoside-diphosphate-sugar epimerase (326 aa, COG0451); 15. Nucleoside-diphosphate-sugar pyrophosphorylase (249 aa, pfam00483); 16. Galactokinase (377 aa, COG0153); 17. Soluble lytic murein transglycosylase (347 aa, COG0741); 18. hypothetical protein (235 aa); 19. Predicted UDP-glucose 6-dehydrogenase (283 aa, COG1004).

The degradation of other components of the plant cell wall, such as pectin, contributes to reduction of plant biomass. Together the ZC1 and ZC2 metagenomes have 584 predicted genes related to pectin degradation, such as pectate lyase (COG 3866), endopolygalacturonase (COG5434) and pectin methylesterase (COG4677). In ZC1 contig 00009.9 (27,919 bp) we found genes encoding these three enzymes along with predicted genes related to carbohydrate metabolism and other functions (Fig. 6). This contig appears to belong to a member of the bacteroidales order (data not shown). Altogether these results provide strong evidence for the notion that at the composting stage when ZC1 was sampled the microbial community has high metabolic potential for complex carbohydrate deconstruction and utilization of released oligosaccharides.

### Putative Lignin-degrading Genes

Aware of the considerable interest in lignin breakdown methods for conversion of lignocellulose into second-generation biofuels and renewable aromatic chemicals [74], we searched for predicted genes related to lignin peroxidases and copper-dependent laccases in the ZC1 and ZC2 metagenomes. These are extracellular enzymes produced by ligninolytic white-rot and brown-rot fungi [75]. As noted above, fungi were essentially absent from ZC1 and ZC2; but several reports have described the ability of bacteria to breakdown lignin [74]. We found 43 (ZC1) and 190 (ZC2) predicted genes coding for iron-dependent peroxidases, which include Dyp-type peroxidases (pfam04261). For instance, the complete coding sequence of a Dyp-type peroxidase found in ZC1, with 307 aa, is 94% identical to a putative dyp-type peroxidase from *Acinetobacter sp.* (GI:389721224) (Figure S2). In ZC2 we identified a dyp-type peroxidase complete coding sequence (318 aa) that is 100% identical to a Dyp-type peroxidase from *Lactobacillus acidipiscis* KCTC 13900 (GI:366090439) (Figure S2). However, neither was predicted to be a secreted enzyme. The Dyp-type peroxidase family appears to contain bifunctional enzymes, with hydrolase or oxygenase, as well as typical peroxidase activities [76]. It has been suggested that secreted bacterial Dyp-type peroxidases may represent the bacterial counterpart of the fungal lignin peroxidases, with examples being the ones produced by the Actinomycetales *Rhodococcus* sp. and *Thermobifida fusca* [77], [78]. On the other hand, both ZC1 and ZC2 metagenomes contain, respectively 224 and 110 sequences encoding genes with similarity to heme-dependent bifunctional catalase-peroxidase (EC:1.11.1.7/EC:1.11.1.6), a family of enzymes recently proposed to contribute to lignin degradation in the Actinomycetales *Amycolatopsis* sp [79]. In ZC1 we found a predicted gene 60% identical to a catalase-peroxidase from *Amycolatopsis* sp (GI: 385676086) (Figure S3). Thus, it appears that ZC1 and ZC2 have the potential for lignin degradation of the compost lignocellulosic biomass. Based on the above observations, we hypothesize that this capability is due to Actinomycetales species present in both microbiomes (Fig. 2).

### Comparison with Seven Other Metagenomes

We compared the two composting microbiomes with seven public metagenomes: benzene-degrading bioreactor, biofuel reactor, compost minireactor, termite hindgut, poplar biomass bioreactor, lake sediment, and rain forest soil. The general features of these metagenomes are listed in Table S5. Among the criteria for selecting these public metagenomes for our comparative analyses were their relatedness to biomass deconstruction environments, whole shotgun sequencing strategy, and annotation of assembled sequences publicly available on IMG/M [80].

**Figure 7:**
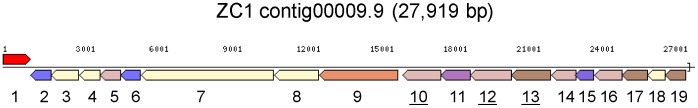
Relative abundance of COG functional categories for ZC1 and ZC2 and seven public metagenomes. Assembled sequence reads were classified into the 25 COG categories designated in Figure 5 and their relative abundances for each metagenome were estimated considering the respective total number of protein coding sequences with function prediction. The public metagenomes included in the comparison are benzene-degrading bioreactor, biofuel reactor, compost minireactor, termite hindgut, poplar biomass bioreactor, lake sediment and soil rain forest, whose features are listed in Table S5. Asterisks indicate statistically significant values.

The COG functional categories overall distribution for the seven public metagenomes reflects the dominance of the Bacteria domain, similarly to what was seen for the ZC1 and ZC2 metagenomes (Fig. 7), even though each individual microbiome composition is quite different. As described above, ZC1 presents a significant abundance of Clostridiales, but Lactobacillales predominate in ZC2 (Fig. 2). The termite hindgut microbiome is enriched in Spirochaetales and Fibrobacterales [37], and the biofuel reactor metagenome is highly enriched in Bacteroidales and Clostridiales (IMG/M unpublished data).

Again here, at the highest level of the COG system, we found general agreement of the distribution in ZC1 and ZC2 compared with the selected seven public metagenomes, but with some differences (Fig. 7). Among the broad differences we highlight the following. In the ZC2, biofuel reactor, and rain forest soil metagenomes COGs belonging to functional category G (Carbohydrate transport and metabolism) are statistically overrepresented compared with the other metagenomes except termite hindgut. The functional category K (Transcription) is also statistically overrepresented in the rain forest soil compared with the other metagenomes. On the other hand, secondary metabolite biosynthesis-related COGs are statistically overrepresented in the compost minireactor, poplar biomass bioreactor and lake sediment metagenomes, but less abundant in the termite hindgut microbiome (Fig. 7, Functional category Q). Also, the termite hindgut metagenome is particularly rich in cell motility COGs in comparison with the other metagenomes (Fig. 7, Functional category N), as has already been noted [81]. Even though functions related to signal transduction mechanisms are enriched in the ZC1 and ZC2 metagenomes as discussed above (Table 3), the other seven metagenomes are even more enriched in this category (Fig. 7, Functional category T).

At deeper levels of the COG system, a comparison of COG functions present in the compost metagenomes and in the seven selected metagenomes revealed a set of 35 and 179 COGs statistically overrepresented respectively in ZC1 (15,623 predicted genes) and ZC2 (76,175 predicted genes) (Table S6). Among these overrepresented COGs are those associated with bacterial efflux pumps (COG 1132 and COG0534), which are abundant within the ZC1 and ZC2 metagenomes, as already noted above. The set of COGs statistically overrepresented in ZC2 with respect to the other seven metagenomes include predicted genes related to fermentation, such as Pyruvate/2-oxoglutarate dehydrogenase complex and L-lactate dehydrogenase, which is consistent for a metagenome in which *Lactobacillus* species predominate. Also, predicted genes related to phosphotransferase system (COG1455, COG1263 and COG1264) and to ABC-type transport systems (Table S6) are overrepresented in the ZC2 metagenome, revealing its high potential for sugar uptake.

The metabolic potential of the ZC1 and ZC2 metagenomes to hydrolyze cellulose, xylan, pectin, as well as proteins is also evident when relative abundance of sequences encoding relevant degradative enzymes is compared with the other seven metagenomes (Table S7). Statistically significant differences in relative abundance for some Enzyme Commission (E.C.) numbers related to those processes were observed. The ZC1 metagenome is enriched in predicted genes encoding cellulase activity (EC:3.2.1.4) and N-acetylmuramoyl-L-alanine amidase (EC:3.5.1.28), while the ZC2 metagenome is enriched in predicted genes encoding membrane alanyl aminopeptidase (EC:3.4.11.2), protein-tyrosine-phosphatase (EC:3.1.3.48), choloylglycine hydrolase (EC:3.5.1.24), lysozyme (EC:3.2.1.17) and Xaa-Pro dipeptidyl-peptidase (EC:3.4.14.11).

**Figure 8:**
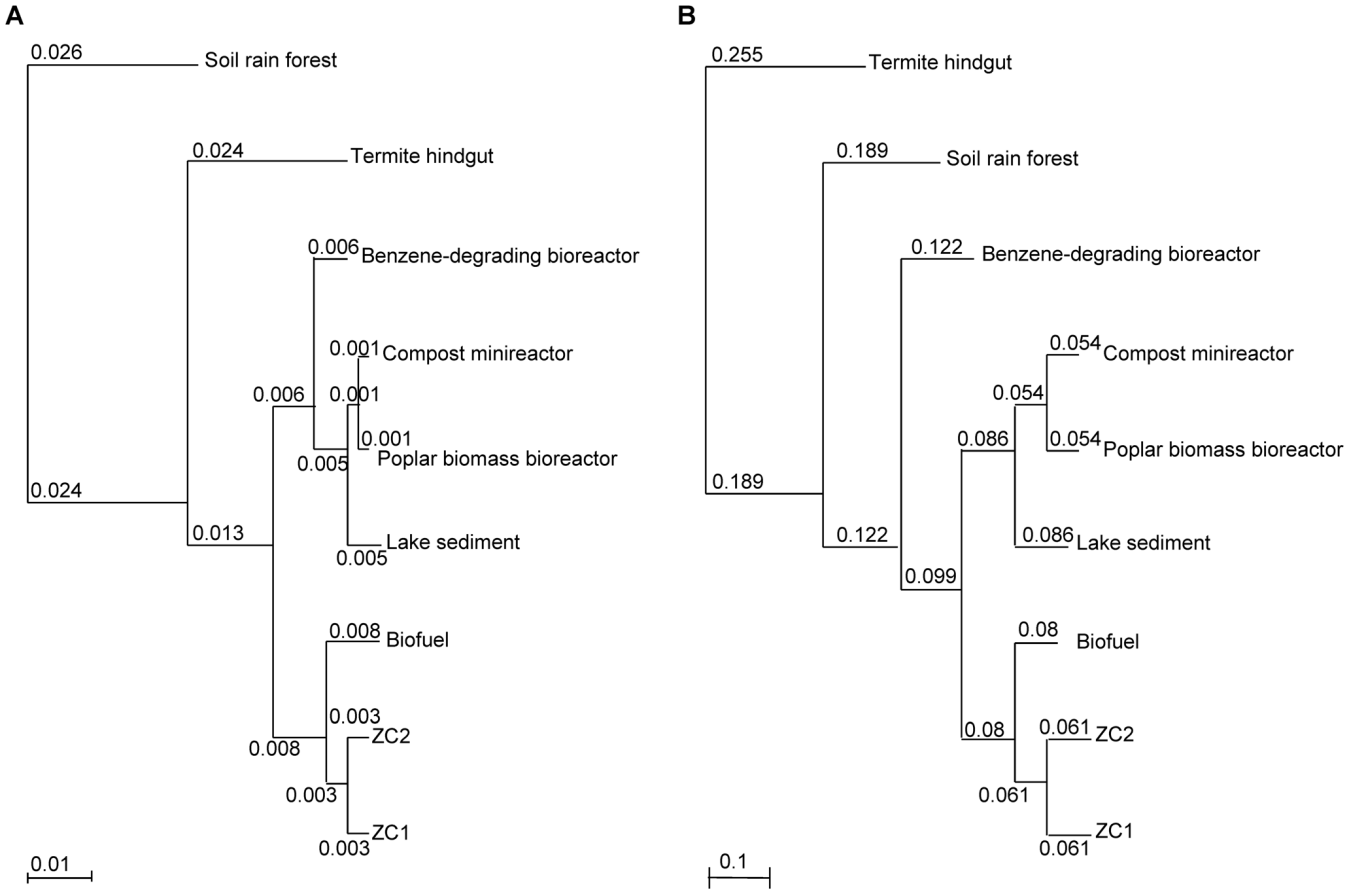
Hierarchical clustering of functional gene groups of ZC1 and ZC2 and seven public metagenomes. (A) Clustering based on COG functional categories; (B) clustering based on COG functions. Hierarchical trees were generated using the “Compare Genomes” tool in IMG/M. Branch lengths are shown.

A hierarchical clustering of functional gene groups based on COG functional categories and on COG functions of ZC1, ZC2 and the seven public metagenomes (Fig. 8) emphasize points made above. In both diagrams ZC1 and ZC2 cluster together, demonstrating their similar functional profile, despite large differences in microbial species composition. In the clustering using the highest COG categories (Fig. 8A), branch lengths are short, giving evidence of the compositional similarity among the metagenomes compared. In the clustering using COG functions (Fig. 8B) we see much longer branch lengths, denoting their specificities.

### Concluding Remarks

Composting is a highly dynamic process involving changing microbial communities that are very efficient in organic matter decomposition. Here, the complexity of this process was analyzed at a detailed level by shotgun metagenomic sequencing. Our results fit well with the current understanding that biomass degradation in composting, including deconstruction of recalcitrant lignocellulose, is fully performed by bacterial enzymes, possibly derived from Clostridiales and Actinomycetales [20], [74]. Although fungi are generally considered the main microbial decomposers of plant material [75], their role in composting is possibly diminished because of frequent anaerobic and thermophilic conditions in semi-static composting processes like the São Paulo Zoo composting operation, similarly to what has been observed in the anaerobic decomposing of poplar wood chips [82]. Our results indicate that cellulose and hemicellulose deconstruction during the composting process appear to be performed by cellulosomal enzymes. Indeed, it has been proposed that the cellulosome is more efficient in degrading complex plant polysaccharides than “free enzymes” produced by aerobic bacteria and fungi [73].

Despite the differences in the phylogenetic profile of the two microbiomes we have analyzed, their overall functional profile is similar. Moreover, we found a general agreement of the Zoo compost metagenomes functional categories distribution in comparison with seven selected metagenomes of biomass deconstruction environments. On the other hand, the organism composition of these microbiomes are quite different, indicating the potential for distinct bacterial communities to provide alternative mechanisms for the same functional purposes. If correct, this suggests that complex microbial environments harbor functional capabilities carried out in novel ways. In support of this we note that a new strategy for lignocellulose degradation has been recently described in yak rumen, which does not involve either cellulosomes or a free-enzyme system [27].

It is also notable that genes encoding proteins related to pectin degradation are present in the Zoo compost metagenomes. Pectin-rich biomass has been considered as an alternative feedstock for biofuel production [83]. Thus, a composting operation such as the one we analyzed here can be considered a rich source for prospection of biomass degradation enzymes. Moreover, continued exploration of complex environments such as composting will foster the discovery of compounds (e.g. antibiotics) and/or mechanisms (e.g. interspecies bacterial communication) relevant to the understanding of how particular environments drive the functional structure of microbial communities.

## Methods

### Sample Collection and DNA Extraction

Two 8 m^3^ concrete chambers ZC1 and ZC2 were established, respectively on 01/26/2011 and 07/21/2009, for composting, following routine procedures at the São Paulo Zoo Park composting facility with minor modifications from a previously described method [84] to attend the needs of a large composting operation. The two cells were fed with similar biosolids composed by shredded tree branches and leaves from the surrounding Atlantic rain forest, plus manure, beddings and food residues from about 400 species of zoo animals (mammals, avian and reptiles), so that both reached a Carbon: Nitrogen ratio of roughly 30∶1. Adequate aerobic conditions were maintained by having air pipes at the bottom of the chamber and by arranging the bio-residues in a way to permit air flowing from bottom to top through shredded tree branches and wood chips. The chambers were watered once a week to maintain proper humidity levels (50–60%) and to avoid excessive heating. Moisture content was estimated by microwave oven drying as previously described [84]. Temperature was measured weekly at five points in each chamber; reported temperatures are averages of the five measures. Over the course of the composting process temperatures in the composting mass oscillated between 50 and 72°C. The compost was thoroughly mixed using a BobCat skid-steer loader around day 40 after temperature dropped below 55°C; immediately after, temperatures climbed back to the 70–72°C range, thus ensuring thermophilic conditions. No undesirable odors were detected during the composting process, indicating that a desirable aerobic level was reached. After ∼90 days the compost material was removed and aged for an additional ∼10 days in windrows.

Samples were collected following the protocol previously described [85], at day 8 of composting from one chamber (Zoo Compost 1, ZC1) and at day 60 of composting from another chamber (Zoo Compost 2, ZC2) which had been aerated 8 days earlier. In brief, each sample of approximately 300 g was made by pooling 5 subsamples taken from 5 points of each compost pile. At the moment of sampling, average temperature was 65.8°C and 67.2°C for ZC1 and ZC2, respectively, and pH was 7.0 for both. Samples were stored at −80°C until DNA extraction. Aliquots of the ZC1 and ZC2 samples were lyophilized and macerated, and approximately 2 g of dried material was used for DNA extraction with MoBio DNA Power Soil kit (MoBio Laboratories, Carlsbad, CA). Some samples (including ZC2, but not ZC1) were pre-treated with lysozyme, Proteinase K and sodium dodecyl sulfate prior to purification with the MoBio kit. The critical step for DNA extraction was the maceration with grinding mortar and pestle, and both ZC1 and ZC2 samples were macerated under the same conditions. Mechanical cell lysing through maceration was shown to be more effective than chemical or enzymatic lysing. Thus we believe it is highly unlikely that enzymatic pre-treatment in the DNA extraction procedure would have favored DNA extraction of selected bacterial groups. DNA purity and concentration was analyzed by spectrophotometric quantification at 260 nm, 280 nm and 230 nm and using Invitrogen’s Quant-iT Picogreen dsDNA BR assay kit. Metagenomic DNA integrity was examined using Agilent Bioanalyser DNA 7500 LabChip.

### Pyrosequencing and Sequence Analysis

The two DNA samples (500 ng) were submitted to pyrosequencing following standard Roche 454 GS FLX Titanium protocols (Roche Applied Science). Shotgun libraries for ZC1 and ZC2 DNA were constructed using GS Titanium Rapid Library Prep Kit and submitted to four sequencing runs. Sequencing reads were quality-filtered and assembled using 454 Newbler assembler software version 2.5.3. The resulting sets of contigs (including singlets) were submitted to the IMG/M annotation pipeline [80]. Unassembled raw reads were submitted to annotation on MG-RAST metagenomics analysis server [40] using their default quality control pipeline.

Microbial composition analyses were performed using MG-RAST best hit classification tool against the databases M5RNA (Non-redundant multisource ribosomal RNA annotation) or RDP (Ribosomal Database Project) available within MG-RAST (version 3.2.4.2) [40] using minimum identity of 98%, maximum e-value cutoff of 10^−30^ and minimum alignment length of 50 bp. Analyses were also done against M5NR (M5 non-redundant protein) using minimum identity of 60%, maximum e-value cutoff of 10^−5^ and minimum alignment length of 50 bp.

Bacterial taxonomy classification and rarefaction were obtained using rRNA-related sequences retrieved from the whole metagenomic sequences data set (4,420 sequences for ZC1 and 5,616 sequences for ZC2, annotated as rRNA-related by MG-RAST) and the Classifier and PYRO pipeline tools in the Ribosomal Database Project [41].

*Lactobacillus* species identification in ZC2 was done by comparing ZC2 reads using BLAST against three different databases. The first was the RDP database of 16S rRNA sequences (version 10) [41]; the second was the NT database from GenBank (downloaded on 6/19/2012); and the third was the M5NR database available within MG-RAST (version 3.2.4.2) [40]. For the RDP and NT databases (searched with BLASTN) we used the following conservative criteria: we only considered alignments with at least 200 positions, at least 98% identity to subject sequences, and comparison results in which a defined *Lactobacillus* species (as opposed to *Lactobacillus* sp.) was the first hit. Moreover, a species assignment was considered positive only when the bit score of the first hit was larger than the bit score of the second hit (hits were sorted on bit score) and when there were at least five different reads witnessing the assignment (for RDP) or at least 50 (for NT). The criteria for species assignment against the M5NR database (searched with BLASTX) were those adopted by the MG-RAST pipeline. In defining the final species tally we considered only our results based on the RDP and NT databases, although we do report the M5NR number of hits as well (in Table S4). We have also used the software Metaphlan [86] to confirm these identifications and to provide abundance figures.

Functional classification and comparative analyses of metagenomes were performed based on COG categories, Pfam family and EC numbers for the metagenomic data sets annotated by IMG/M pipeline [80], using the function comparison tool considering its statistical parameters (binomial test). For all tests of statistical overrepresentation we used a maximum *p-*value of 0.05.

### Protein Sequence Comparison and Alignments

Protein-coding gene sequences retrieved from IMG/M were further compared against the NR database of GenBank [87] using BLAST [88] with maximum e-value 10^−5^ and aligned to orthologs using ClustalW [89].

### Hierarchical Clustering

Hierarchical clustering was performed using a matrix of the number of reads assigned to COGs from each metagenome and was generated with the “Compare Genomes” tool in IMG/M [80], which uses uncentered correlation as distance measure and pairwise single-linkage clustering.

### Sequence Data Submission

Datasets are publicly available on IMG/M (ZC1: Taxon Object ID 2209111003; ZC2: Taxon Object ID 2199352030) and MG-RAST (ZC1: ID 4479361.3; ZC2 ID 4479944.3).

## Supporting Information

Figure S1 **Alignment of two ZC1 sequences classified with COG1363 function with a** ***Clostridium thermocellum*** **cellulase M.** Sequences ZC1_1363_1 (349 amino acids) and ZC1_1363_2 (345 amino acids) were aligned to *C. thermocellum* (Ct) cellulase M (GI: 1097207) using Clustal W 2.1.
(TIF)

Figure S2 **Dyp-type peroxidase sequences from ZC1 and ZC2 metagenomes.** Alignment of a dyp-type peroxidase sequence from ZC1 and ZC2 metagenomes with homologs from *Acinetobacter* sp (GI:389721224) and *Lactobacillus acidipiscis* KCTC 13900 (GI:366090439), using Clustal W 2.1
(TIF)

Figure S3 **Heme-dependent bifunctional catalase-peroxidase from ZC1 metagenome.** Alignment of a heme-dependent bifunctional catalase-peroxidase (EC:1.11.1.7/EC:1.11.1.6) from *Amycolatopsis* sp (GI: 385676086) with a homolog from the ZC1 metagenome, using Clustal W 2.1.
(TIF)

Table S1 **Domain distribution on Zoo Compost Samples.**
(XLSX)

Table S2 **Relative abundance of bacterial orders found in ZC1 and ZC2 according RDP and M5NR databases analyses.**
(XLSX)

Table S3 **Relative abundance of bacterial genera found in ZC1 and ZC2 according RDP databases analyses.**
(XLSX)

Table S4 **Diversity of** ***Lactobacillus*** **in ZC2.**
(XLSX)

Table S5 **General features of selected metagenomes for functional comparison.**
(XLSX)

Table S6 **List of the COG functions statistically overabundant in ZC1 and ZC2 against the seven metagenomes selected for comparison.**
(XLSX)

Table S7 **Relative abundance of sequences encoding selected enzymes in nine metagenomes.**
(XLSX)

## References

[1] Ryckeboer J, Mergaert J, Vaes K, Klammer S, De Clercq D, et al (2003) A survey of bacteria and fungi occurring during composting and self-heating processes. Annals of Microbiology 53: 349–410.

[2] Ishii K, Takii S (2003) Comparison of microbial communities in four different composting processes as evaluated by denaturing gradient gel electrophoresis analysis. Journal of Applied Microbiology 95: 109–119. 12807460 10.1046/j.1365-2672.2003.01949.x

[3] Steger K, Eklind Y, Olsson J, Sundh I (2005) Microbial community growth and utilization of carbon constituents during thermophilic composting at different oxygen levels. Microbial Ecology 50: 163–171. 16184337 10.1007/s00248-004-0139-y

[4] Takebayashi S, Narihiro T, Fujii Y, Hiraishi A (2007) Water availability is a critical determinant of a population shift from Proteobacteria to Actinobacteria during start-up operation of mesophilic fed-batch composting. Microbes and Environments 22: 279–289.

[5] Vargas-Garcia MC, Suarez-Estrella F, Lopez MJ, Moreno J (2010) Microbial population dynamics and enzyme activities in composting processes with different starting materials. Waste Management 30: 771–778. 20096556 10.1016/j.wasman.2009.12.019

[6] Partanen P, Hultman J, Paulin L, Auvinen P, Romantschuk M (2010) Bacterial diversity at different stages of the composting process. BMC Microbiology 10: 94. 20350306 10.1186/1471-2180-10-94 PMC2907838

[7] Kumar S (2011) Composting of municipal solid waste. Critical Reviews in Biotechnology 31: 112–136. 20854128 10.3109/07388551.2010.492207

[8] Peters S, Koschinsky S, Schwieger F, Tebbe CC (2000) Succession of microbial communities during hot composting as detected by PCR-single-strand-conformation polymorphism-based genetic profiles of small-subunit rRNA genes. Applied and Environmental Microbiology 66: 930–936. 10698754 10.1128/aem.66.3.930-936.2000 PMC91925

[9] Alfreider A, Peters S, Tebbe CC, Rangger A, Insam H (2002) Microbial community dynamics during composting of organic matter as determined by 16S ribosomal DNA analysis. Compost Science & Utilization 10: 303–312.

[10] Steger K, Sjogren AM, Jarvis A, Jansson JK, Sundh I (2007) Development of compost maturity and Actinobacteria populations during full-scale composting of organic household waste. Journal of Applied Microbiology 103: 487–498. 17650210 10.1111/j.1365-2672.2006.03271.x

[11] Guo Y, Zhu N, Zhu S, Deng C (2007) Molecular phylogenetic diversity of bacteria and its spatial distribution in composts. Journal of Applied Microbiology 103: 1344–1354. 17897238 10.1111/j.1365-2672.2007.03367.x

[12] Franke-Whittle IH, Knapp BA, Fuchs J, Kaufmann R, Insam H (2009) Application of COMPOCHIP microarray to investigate the bacterial communities of different composts. Microbial Ecology 57: 510–521. 18818861 10.1007/s00248-008-9435-2

[13] Anastasi A, Varese GC, Marchisio VF (2005) Isolation and identification of fungal communities in compost and vermicompost. Mycologia 97: 33–44. 16389954 10.3852/mycologia.97.1.33

[14] Hultman J, Vasara T, Partanen P, Kurola J, Kontro MH, et al (2010) Determination of fungal succession during municipal solid waste composting using a cloning-based analysis. Journal of Applied Microbiology 108: 472–487. 19656238 10.1111/j.1365-2672.2009.04439.x

[15] Bent SJ, Forney LJ (2008) The tragedy of the uncommon: understanding limitations in the analysis of microbial diversity. The ISME Journal 2: 689–695. 18463690 10.1038/ismej.2008.44

[16] Hong SH, Bunge J, Leslin C, Jeon S, Epstein SS (2009) Polymerase chain reaction primers miss half of rRNA microbial diversity. The ISME Journal 3: 1365–1373. 19693101 10.1038/ismej.2009.89

[17] van Elsas JD, Boersma FGH (2011) A review of molecular methods to study the microbiota of soil and the mycosphere. European Journal of Soil Biology 47: 77–87.

[18] Gonzalez JM, Portillo MC, Belda-Ferre P, Mira A (2012) Amplification by PCR Artificially Reduces the Proportion of the Rare Biosphere in Microbial Communities. PloS ONE 7: e29973. 22253843 10.1371/journal.pone.0029973 PMC3256211

[19] Lombard N, Prestat E, van Elsas JD, Simonet P (2011) Soil-specific limitations for access and analysis of soil microbial communities by metagenomics. FEMS Microbiology Ecology 78: 31–49. 21631545 10.1111/j.1574-6941.2011.01140.x

[20] Allgaier M, Reddy A, Park JI, Ivanova N, D’haeseleer P, et al (2010) Targeted discovery of glycoside hydrolases from a switchgrass-adapted compost community. PloS ONE 5: e8812. 20098679 10.1371/journal.pone.0008812 PMC2809096

[21] Shokralla S, Spall JL, Gibson JF, Hajibabaei M (2012) Next-generation sequencing technologies for environmental DNA research. Molecular Ecology 21: 1794–1805. 22486820 10.1111/j.1365-294X.2012.05538.x

[22] Thomas T, Gilbert J, Meyer F (2012) Metagenomics - a guide from sampling to data analysis. Microbial Informatics and Experimentation 2: 3. 22587947 10.1186/2042-5783-2-3 PMC3351745

[23] Simon C, Daniel R (2009) Achievements and new knowledge unraveled by metagenomic approaches. Applied Microbiology and Biotechnology 85: 265–276. 19760178 10.1007/s00253-009-2233-z PMC2773367

[24] Delmont TO, Malandain C, Prestat E, Larose C, Monier JM, et al (2011) Metagenomic mining for microbiologists. The ISME Journal 5: 1837–1843. 21593798 10.1038/ismej.2011.61 PMC3223302

[25] Brulc JM, Antonopoulos DA, Miller MEB, Wilson MK, Yannarell AC, et al (2009) Gene-centric metagenomics of the fiber-adherent bovine rumen microbiome reveals forage specific glycoside hydrolases. Proceedings of the National Academy of Sciences of the United States of America 106: 1948–1953. 19181843 10.1073/pnas.0806191105 PMC2633212

[26] Hess M, Sczyrba A, Egan R, Kim TW, Chokhawala H, et al (2011) Metagenomic discovery of biomass-degrading genes and genomes from cow rumen. Science 331: 463–467. 21273488 10.1126/science.1200387

[27] Dai X, Zhu YX, Luo YF, Song L, Liu D, et al (2012) Metagenomic insights into the fibrolytic microbiome in yak rumen. PloS ONE 7: e40430. 22808161 10.1371/journal.pone.0040430 PMC3396655

[28] Gladden JM, Allgaier M, Miller CS, Hazen TC, VanderGheynst JS, et al (2011) Glycoside hydrolase activities of thermophilic bacterial consortia adapted to switchgrass. Applied and Environmental Microbiology 77: 5804–5812. 21724886 10.1128/AEM.00032-11 PMC3165268

[29] Dougherty MJ, D’Haeseleer P, Hazen TC, Simmons BA, Adams PD, et al (2012) Glycoside hydrolases from a targeted compost metagenome, activity-screening and functional characterization. BMC Biotechnology 12: 38. 22759983 10.1186/1472-6750-12-38 PMC3477009

[30] Curtis TP, Sloan WT, Scannell JW (2002) Estimating prokaryotic diversity and its limits. Proceedings of the National Academy of Sciences of the United States of America 99: 10494–10499. 12097644 10.1073/pnas.142680199 PMC124953

[31] Torsvik V, Ovreas L, Thingstad TF (2002) Prokaryotic diversity–magnitude, dynamics, and controlling factors. Science 296: 1064–1066. 12004116 10.1126/science.1071698

[32] Schloss PD, Handelsman J (2006) Toward a census of bacteria in soil. PLoS Computational Biology 2: e92. 16848637 10.1371/journal.pcbi.0020092 PMC1513271

[33] Dillon RJ, Dillon VM (2004) The gut bacteria of insects: Nonpathogenic interactions. Annual Review of Entomology 49: 71–92. 10.1146/annurev.ento.49.061802.123416 14651457

[34] Gill SR, Pop M, Deboy RT, Eckburg PB, Turnbaugh PJ, et al (2006) Metagenomic analysis of the human distal gut microbiome. Science 312: 1355–1359. 16741115 10.1126/science.1124234 PMC3027896

[35] Ley RE, Hamady M, Lozupone C, Turnbaugh PJ, Ramey RR, et al (2008) Evolution of mammals and their gut microbes. Science 320: 1647–1651. 18497261 10.1126/science.1155725 PMC2649005

[36] DeAngelis KM, Gladden JM, Allgaier M, D’haeseleer P, Fortney JL, et al (2010) Strategies for enhancing the effectiveness of metagenomic-based enzyme discovery in lignocellulolytic microbial communities. Bioenergy Research 3: 146–158.

[37] Warnecke F, Luginbuhl P, Ivanova N, Ghassemian M, Richardson TH, et al (2007) Metagenomic and functional analysis of hindgut microbiota of a wood-feeding higher termite. Nature 450: 560–565. 18033299 10.1038/nature06269

[38] Hollister EB, Forrest AK, Wilkinson HH, Ebbole DJ, Malfatti SA, et al (2010) Structure and dynamics of the microbial communities underlying the carboxylate platform for biofuel production. Applied Microbiology and Biotechnology 88: 389–399. 20676626 10.1007/s00253-010-2789-7

[39] Ramette A (2007) Multivariate analyses in microbial ecology. FEMS Microbiology Ecology 62: 142–160. 17892477 10.1111/j.1574-6941.2007.00375.x PMC2121141

[40] Meyer F, Paarmann D, D’Souza M, Olson R, Glass EM, et al (2008) The metagenomics RAST server - a public resource for the automatic phylogenetic and functional analysis of metagenomes. BMC Bioinformatics 9: 386. 18803844 10.1186/1471-2105-9-386 PMC2563014

[41] Cole JR, Wang Q, Cardenas E, Fish J, Chai B, et al (2009) The Ribosomal Database Project: improved alignments and new tools for rRNA analysis. Nucleic Acids Research 37: D141–D145. 19004872 10.1093/nar/gkn879 PMC2686447

[42] Steger K, Jarvis A, Vasara T, Romantschuk M, Sundh I (2007) Effects of differing temperature management on development of Actinobacteria populations during composting. Research in Microbiology 158: 617–624. 17683913 10.1016/j.resmic.2007.05.006

[43] Dees PM, Ghiorse WC (2001) Microbial diversity in hot synthetic compost as revealed by PCR-amplified rRNA sequences from cultivated isolates and extracted DNA. FEMS Microbiology Ecology 35: 207–216. 11295460 10.1111/j.1574-6941.2001.tb00805.x

[44] Klammer S, Knapp B, Insam H, Dell’Abate MT, Ros M (2008) Bacterial community patterns and thermal analyses of composts of various origins. Waste Management & Research 26: 173–187. 18578156 10.1177/0734242X07084113

[45] Wang Q, Garrity GM, Tiedje JM, Cole JR (2007) Naive Bayesian classifier for rapid assignment of rRNA sequences into the new bacterial taxonomy. Applied and Environmental Microbiology 73: 5261–5267. 17586664 10.1128/AEM.00062-07 PMC1950982

[46] Schloss PD, Hay AG, Wilson DB, Walker LP (2003) Tracking temporal changes of bacterial community fingerprints during the initial stages of composting. FEMS Microbiology Ecology 46: 1–9. 19719577 10.1016/S0168-6496(03)00153-3

[47] Atkinson CF, Jones DD, Gauthier JJ (1996) Putative anaerobic activity in aerated composts. Journal of Industrial Microbiology 16: 182–188.

[48] Lynd LR, Weimer PJ, van Zyl WH, Pretorius IS (2002) Microbial cellulose utilization: Fundamentals and biotechnology. Microbiology and Molecular Biology Reviews 66: 506–577. 12209002 10.1128/MMBR.66.3.506-577.2002 PMC120791

[49] Yi J, Wu HY, Wu J, Deng CY, Zheng R, et al (2012) Molecular phylogenetic diversity of Bacillus community and its temporal-spatial distribution during the swine manure of composting. Applied Microbiology and Biotechnology 93: 411–421. 21701982 10.1007/s00253-011-3425-x

[50] Kato S, Haruta S, Cui ZJ, Ishii M, Igarashi Y (2004) Effective cellulose degradation by a mixed-culture system composed of a cellulolytic Clostridium and aerobic non-cellulolytic bacteria. FEMS Microbiology Ecology 51: 133–142. 16329862 10.1016/j.femsec.2004.07.015

[51] Bugg TDH, Ahmad M, Hardiman EM, Rahmanpour R (2011) Pathways for degradation of lignin in bacteria and fungi. Natural Product Reports 28: 1883–1896. 21918777 10.1039/c1np00042j

[52] Tuomela M, Vikman M, Hatakka A, Itavaara M (2000) Biodegradation of lignin in a compost environment: a review. Bioresource Technology 72: 169–183.

[53] Yu H, Zeng GM, Huang HL, Xi XM, Wang RY, et al (2007) Microbial community succession and lignocellulose degradation during agricultural waste composting. Biodegradation 18: 793–802. 17308882 10.1007/s10532-007-9108-8

[54] Rastogi G, Bhalla A, Adhikari A, Bischoff KM, Hughes SR, et al (2010) Characterization of thermostable cellulases produced by Bacillus and Geobacillus strains. Bioresource Technology 101: 8798–8806. 20599378 10.1016/j.biortech.2010.06.001

[55] Gu Y, Ding Y, Ren C, Sun Z, Rodionov DA, et al (2010) Reconstruction of xylose utilization pathway and regulons in Firmicutes. BMC Genomics 11: 255. 20406496 10.1186/1471-2164-11-255 PMC2873477

[56] Gihring TM, Green SJ, Schadt CW (2012) Massively parallel rRNA gene sequencing exacerbates the potential for biased community diversity comparisons due to variable library sizes. Environmental Microbiology 14: 285–290. 21923700 10.1111/j.1462-2920.2011.02550.x

[57] Aoshima M, Pedro MS, Haruta S, Ding LX, Fukada T, et al (2001) Analyses of microbial community within a composter operated using household garbage with special reference to the addition of soybean oil. Journal of Bioscience and Bioengineering 91: 456–461. 16233022 10.1263/jbb.91.456

[58] Hemmi H, Shimoyama T, Nakayama T, Hoshi K, Nishino T (2004) Molecular biological analysis of microflora in a garbage treatment process under thermoacidophilic conditions. Journal of Bioscience and Bioengineering 97: 119–126. 16233602 10.1016/S1389-1723(04)70178-4

[59] Adams JD, Frostick LE (2009) Analysis of bacterial activity, biomass and diversity during windrow composting. Waste Management 29: 598–605. 18977649 10.1016/j.wasman.2008.06.037

[60] Azadnia P, Zamani MH, Ghasemi SA, Babaki AK, Jashni MK, et al (2011) Isolation and identification of thermophilic Lactobacilli from traditional yoghurts of tribes of Kazerun. Journal of Animal and Veterinary Advances 10: 774–776.

[61] Dobson A, Cotter PD, Ross RP, Hill C (2012) Bacteriocin production: a probiotic trait? Applied and Environmental Microbiology 78: 1–6. 22038602 10.1128/AEM.05576-11 PMC3255625

[62] Singh S, Goswami P, Singh R, Heller KJ (2009) Application of molecular identification tools for Lactobacillus, with a focus on discrimination between closely related species: A review. Lwt-Food Science and Technology 42: 448–457.

[63] Lukjancenko O, Ussery DW, Wassenaar TM (2012) Comparative genomics of Bifidobacterium, Lactobacillus and related probiotic genera. Microbial Ecology 63: 651–673. 22031452 10.1007/s00248-011-9948-y PMC3324989

[64] Scheirlinck I, Van der Meulen R, Van Schoor A, Huys G, Vandamme P, et al (2007) Lactobacillus crustorum sp nov., isolated from two traditional Belgian wheat sourdoughs. International Journal of Systematic and Evolutionary Microbiology 57: 1461–1467. 17625176 10.1099/ijs.0.64836-0

[65] Tanasupawat S, Shida O, Okada S, Komagata K (2000) Lactobacillus acidipiscis sp nov and Weissella thailandensis sp nov., isolated from fermented fish in Thailand. International Journal of Systematic and Evolutionary Microbiology 50: 1479–1485. 10939653 10.1099/00207713-50-4-1479

[66] Canchaya C, Claesson MJ, Fitzgerald GF, van Sinderen D, O’Toole PW (2006) Diversity of the genus Lactobacillus revealed by comparative genomics of five species. Microbiology-SGM 152: 3185–3196. 10.1099/mic.0.29140-0 17074890

[67] Ventura M, O’Flaherty S, Claesson MJ, Turroni F, Klaenhammer TR, et al (2009) Genome-scale analyses of health-promoting bacteria: probiogenomics. Nature Reviews Microbiology 7: 61–71. 19029955 10.1038/nrmicro2047

[68] Endo A, Futagawa-Endo Y, Dicks LMT (2010) Diversity of Lactobacillus and Bifidobacterium in feces of herbivores, omnivores and carnivores. Anaerobe 16: 590–596. 21034840 10.1016/j.anaerobe.2010.10.005

[69] Tatusov RL, Fedorova ND, Jackson JD, Jacobs AR, Kiryutin B, et al (2003) The COG database: an updated version includes eukaryotes. BMC Bioinformatics 4: 41. 12969510 10.1186/1471-2105-4-41 PMC222959

[70] Piddock LJV (2006) Multidrug-resistance efflux pumps - not just for resistance. Nature Reviews Microbiology 4: 629–636. 16845433 10.1038/nrmicro1464

[71] Cottrell MT, Yu LY, Kirchman DL (2005) Sequence and expression analyses of Cytophaga-like hydrolases in a Western arctic metagenomic library and the Sargasso seat. Applied and Environmental Microbiology 71: 8506–8513. 16332841 10.1128/AEM.71.12.8506-8513.2005 PMC1317373

[72] Demain AL, Newcomb M, Wu JHD (2005) Cellulase, clostridia, and ethanol. Microbiology and Molecular Biology Reviews 69: 124–154. 15755956 10.1128/MMBR.69.1.124-154.2005 PMC1082790

[73] Fontes CMGA, Gilbert HJ (2010) Cellulosomes: Highly efficient nanomachines designed to deconstruct plant cell wall complex carbohydrates. Annual Review of Biochemistry 79: 655–681. 10.1146/annurev-biochem-091208-085603 20373916

[74] Bugg TDH, Ahmad M, Hardiman EM, Singh R (2011) The emerging role for bacteria in lignin degradation and bio-product formation. Current Opinion in Biotechnology 22: 394–400. 21071202 10.1016/j.copbio.2010.10.009

[75] Sanchez C (2009) Lignocellulosic residues: Biodegradation and bioconversion by fungi. Biotechnol Advances 27: 185–194. 10.1016/j.biotechadv.2008.11.001 19100826

[76] Sugano Y (2009) DyP-type peroxidases comprise a novel heme peroxidase family. Cellular and Molecular Life Sciences 66: 1387–1403. 19099183 10.1007/s00018-008-8651-8 PMC11131544

[77] Roberts JN, Singh R, Grigg JC, Murphy MEP, Bugg TDH, et al (2011) Characterization of dye-decolorizing peroxidases from Rhodococcus jostii RHA1. Biochemistry 50: 5108–5119. 21534572 10.1021/bi200427h

[78] Ahmad M, Taylor CR, Pink D, Burton K, Eastwood D, et al (2010) Development of novel assays for lignin degradation: comparative analysis of bacterial and fungal lignin degraders. Molecular Biosystems 6: 815–821. 20567767 10.1039/b908966g

[79] Brown ME, Walker MC, Nakashige TG, Iavarone AT, Chang MCY (2011) Discovery and characterization of heme enzymes from unsequenced Bacteria: Application to microbial lignin degradation. Journal of the American Chemical Society 133: 18006–18009. 21671563 10.1021/ja203972q

[80] Markowitz VM, Ivanova NN, Szeto E, Palaniappan K, Chu K, et al (2008) IMG/M: a data management and analysis system for metagenomes. Nucleic Acids Research 36: D534–538. 17932063 10.1093/nar/gkm869 PMC2238950

[81] Lamendella R, Domingo JW, Ghosh S, Martinson J, Oerther DB (2011) Comparative fecal metagenomics unveils unique functional capacity of the swine gut. BMC Microbiology 11: 103. 21575148 10.1186/1471-2180-11-103 PMC3123192

[82] van der Lelie D, Taghavi S, McCorkle SM, Li LL, Malfatti SA, et al (2012) The metagenome of an anaerobic microbial community decomposing poplar wood chips. PloS ONE 7: e36740. 22629327 10.1371/journal.pone.0036740 PMC3357426

[83] Edwards MC, Doran-Peterson J (2012) Pectin-rich biomass as feedstock for fuel ethanol production. Applied Microbiology and Biotechnology 95: 565–575. 22695801 10.1007/s00253-012-4173-2 PMC3396330

[84] Rynk R, van de Kamp M, Willson GB, Singley ME, Richard TL, et al.. (1992) On-Farm Composting Handbook, Northeast Regional Agricultural Engineering Service – Cooperative Extension; Rynk R, editor. Ithaca, NY: Northeast Regional Agricultural Engineering Service. 186 p.

[85] Bitencourt ALV, Vallim MA, Maia D, Spinelli R, Angeloni R, et al (2010) Core sampling test in large-scale compost cells for microorganism isolation. African Journal of Microbiology Research 4: 1631–1634.

[86] Segata N, Waldron L, Ballarini A, Narasimhan V, Jousson O, et al (2012) Metagenomic microbial community profiling using unique clade-specific marker genes. Nat Methods 9: 811–814. 22688413 10.1038/nmeth.2066 PMC3443552

[87] Sayers EW, Barrett T, Benson DA, Bolton E, Bryant SH, et al (2012) Database resources of the National Center for Biotechnology Information. Nucleic Acids Research 40: D13–25. 22140104 10.1093/nar/gkr1184 PMC3245031

[88] Altschul SF, Madden TL, Schaffer AA, Zhang J, Zhang Z, et al (1997) Gapped BLAST and PSI-BLAST: a new generation of protein database search programs. Nucleic Acids Research 25: 3389–3402. 9254694 10.1093/nar/25.17.3389 PMC146917

[89] Larkin MA, Blackshields G, Brown NP, Chenna R, McGettigan PA, et al (2007) Clustal W and clustal X version 2.0. Bioinformatics 23: 2947–2948. 17846036 10.1093/bioinformatics/btm404

