# Correction: Metagenomic Analysis of a Tropical Composting Operation at the São Paulo Zoo Park Reveals Diversity of Biomass Degradation Functions and Organisms

**DOI:** 10.1371/annotation/2cca811e-d45b-4854-a420-d0405309ef43

**Published:** 2013-06-27

**Authors:** Layla Farage Martins, Luciana Principal Antunes, Renata C. Pascon, Julio Cezar Franco de Oliveira, Luciano A. Digiampietri, Deibs Barbosa, Bruno Malveira Peixoto, Marcelo A. Vallim, Cristina Viana-Niero, Eric H. Ostroski, Guilherme P. Telles, Zanoni Dias, João Batista da Cruz, Luiz Juliano, Sergio Verjovski-Almeida, Aline Maria da Silva, João Carlos Setubal

The labels and legends for Figures 6 and 7 were incorrectly switched. 

The label and legend appearing under Figure 6 belong under Figure 7, and the legend and label appearing under Figure 7 belong under Figure 6. The figures themselves are in correct order.

